# miR-20a-5p contributes to osteogenic differentiation of human dental pulp stem cells by regulating BAMBI and activating the phosphorylation of Smad5 and p38

**DOI:** 10.1186/s13287-021-02501-8

**Published:** 2021-07-22

**Authors:** Xiao Cen, Xuefeng Pan, Bo Zhang, Wei Huang, Fang Pei, Tao Luo, Xinqi Huang, Jun Liu, Zhihe Zhao

**Affiliations:** 1 State Key Laboratory of Oral Diseases & National Clinical Research Center for Oral Diseases, West China Hospital of Stomatology, Sichuan University, No. 14, 3rd Section, South Renmin Road, Chengdu, 610041 Sichuan China; 2 Department of Temporomandibular Joint, West China Hospital of Stomatology, Sichuan University, Chengdu, China; 3 Department of Orthodontics, West China Hospital of Stomatology, Sichuan University, Chengdu, China; 4 Department of Stomatology, Sichuan Cancer Hospital & Institute, Sichuan Cancer Center, School of Medicine, University of Electronic Science and Technology of China, Chengdu, China

**Keywords:** miR-20a-5p, Human dental pulp stem cells, Osteogenesis, BAMBI

## Abstract

**Background:**

Human dental pulp stem cells (hDPSCs) are the preferable choice of seed cells for craniomaxillofacial bone tissue regeneration. As a member of the miR-17-92 cluster, miR-20a-5p functions as an important regulator during bone remodeling. This study aimed to investigate the roles and mechanisms of miR-20a-5p during osteogenesis of hDPSCs.

**Methods:**

Quantitative reverse transcription-polymerase chain reaction (qRT-PCR) was conducted to determine the expression of miR-20a-5p during osteogenesis of hDPSCs. We interfered with the expression of miR-20a-5p in hDPSCs to clarify the function of miR-20a-5p on osteogenesis both in vitro and vivo. Direct bind sites between miR-20a-5p and BAMBI were confirmed by dual-luciferase reporter assay, and the underlying mechanisms were investigated with cell co-transfections.

**Results:**

The expression of miR-20a-5p was showed to be upregulated during osteogenesis of hDPSCs. Inhibition of miR-20a-5p could weaken the intensity of ALP/ARS staining and downregulate the expression of mRNAs and proteins of osteogenic markers, while overexpression of miR-20a-5p could enhance the intensity of ALP/ARS staining and the expression of osteogenic markers. Both micro-CT reconstruction images and histological results showed that miR-20a-5p could promote the regeneration of calvarial defects. miR-20a-5p directly targeted bone morphogenetic protein and activin membrane-bound inhibitor (BAMBI), and the latter one was an inhibitor of hDPSC osteogenesis. Silencing BAMBI partially reversed the suppression effect of miR-20a-5p knockdown on osteogenesis. Phosphorylation of Smad5 and p38 was decreased when miR-20a-5p was silenced, whereas p-Smad5 and p-p38 were upregulated when miR-20a-5p was overexpressed or BAMBI was silenced.

**Conclusions:**

It is demonstrated that miR-20a-5p functioned as a regulator of BAMBI to activate the phosphorylation of Smad5 and p38 during osteogenic differentiation of hDPSCs.

**Supplementary Information:**

The online version contains supplementary material available at 10.1186/s13287-021-02501-8.

## Background

As members of craniomaxillofacial tissues, human dental pulp stem cells (hDPSCs) are originated from the neural crest. hDPSCs have multidirectional differentiation potentials, which could differentiate into osteogenic, adipogenic, chondrogenic, and neurogenic lineage cells [1, 2]. Compared with mesenchymal stem cells (MSCs) derived from other tissues of humans, hDPSCs possess some advantages. The increasing expression of key pluripotency factors, including OCT4, SOX2, and KLF4, is found in hDPSCs, which suggest the superior property of multidirectional differentiation [3–8]. It is reported that hDPSCs maintain stronger osteogenic activities when compared with the MSCs derived from the bone marrow [9]. Moreover, they could be isolated in a noninvasive and safer method without ethical controversy and possess lower immunogenicity [10, 11]. Therefore, it is suggested hDPSCs could be the preferable choice of seed cells for bone regeneration, while the mechanism on osteogenic differentiation of hDPSCs remains to be clarified.

MicroRNAs (miRNAs) are endogenous single-strand noncoding RNAs with 20–25 nucleotides, which could regulate the expression levels of target genes on the post-transcriptional level [12]. They could function in the translation or enhance the degradation of their target genes directly [13–15]. Emerging evidence has demonstrated their vital functions in numerous biological processes, such as cell proliferation, cell apoptosis, cell differentiation, and antiviral defense [16]. As a member of the miR-17~92 gene cluster, miR-20-5p has been showed to participate in a variety of cellular activities. For instance, miR-20a-5p is involved in myoblast differentiation [17], pulmonary surfactant gene expression [18], angiogenesis [19], immunomodulation [20], and tumorigenesis [21–23]. Regarding the roles of miR-20a-5p in the bone metabolism, previous studies show miR-20a-5p could promote osteogenic differentiation of hMSCs and human adipose-derived stem cells (hASCs) [24, 25], and miR-20a-5p could target PPARγ to regulate osteoclastogenesis of THP-1 cells [26].

This study was aimed to determine whether miR-20a-5p regulated osteogenesis of hDPSCs and further explored the potential mechanism of its regulatory roles. It could shed new lights for DPSC-based bone regeneration.

## Materials and methods

### Cell cultures

The hDPSCs were isolated and cultured as described previously [27]. After getting informed consent from all participants and parents/legally authorized representatives of minors, the premolars were collected from donors (12 to 14 years old) who underwent teeth extraction because of orthodontic needs. The dental pulps were isolated and digested in a solution supplemented with 1 mg/ml type I collagenase (Gibco) in a shaking bath for 1 h at 37 °C. The treated pulp tissue was transferred into a flask containing low glucose Dulbecco’s modified Eagle’s medium (DMEM; Gibco), 1% penicillin-streptomycin (Gibco), and 10% fetal bovine serum (FBS, Gibco). The hDPSCs were cultured in incubation at 37 °C, and the medium was replaced every 3 days. The hDPSCs at passage 4 were used for subsequent experiments.

### Osteogenic differentiation

For the induction of osteogenic differentiation, the hDPSCs were cultured in the osteogenic induction medium consisted of DMEM, 100nM dexamethasone (Sigma), 10 mM β-glycerophosphate (Sigma), and 50 μg/mL ascorbic acid (Sigma).

### Flow cytometric analysis

The hDPSCs were harvested in Dulbecco’s phosphate-buffered saline (DPBS, Thermo Scientific) and incubated for 30 min at 4 °C with FITC-conjugated anti-human CD29, FITC-conjugated anti-human CD44, FITC-conjugated anti-human CD34, and FITC-conjugated anti-human CD45 (BD Biosciences) protected from light. The samples were tested in the flow cytometer (Beckman Coulter, FC500, FL, USA), and the data were analyzed by FlowJo software (Tree Star, San Carlos, CA, USA).

### Cell transfection

Lentiviruses containing miR-20a-5p mimics (miR-20a-5p), miR-20a-5p sponge (anti-miR-20a-5p), and negative control of miRNA (miR-NC) with green fluorescent protein (GFP) were purchased from Hanbio. Cell transfection with miR-20a-5p, anti-miR-20a-5p, and NC at a multiplicity of infection (MOI) of 50 was implemented with 5 mg/ml polybrene (Invitrogen). The hDPSCs were exposed to viral supernatant for 24 h, followed by treated with 1 μg/ml puromycin (Sigma). Then, the cells were washed with phosphate-buffered saline (PBS) and cultured in a general medium. The proportion of GFP-positive cells was observed with a microscope (IX71; Olympus, Tokyo, Japan) to calculate transfection efficiency.

The RNA oligoribonucleotides including the small interfering RNAs (siRNAs) targeting bone morphogenetic protein and activin membrane-bound inhibitor (BAMBI) (si-BAMBI) and NC were designed and synthesized by GenePharma. The hDPSCs (2×10^5^ per well) were seeded on 24-well plates and cultured with DMEM, and they were transfected using Lipofectamine 3000 Reagent (Invitrogen) after 70% confluency. The primers of these RNA oligoribonucleotides were listed in Table S1.

### Alkaline phosphatase (ALP) staining and activity

The hDPSCs were cultured in an osteogenic induction medium for 7 days and used for ALP staining and ALP activity. ALP staining was conducted with the Alkaline Phosphatase Assay Kit (Beyotime). Briefly, cells were washed with PBS and then fixed in citrate solution for 30 s, followed by staining with a solution of FRV alkaline, naphthol AS-BI alkaline, and sodium nitrite for 15 min protected from light. To calculate the ALP activity, the cells were washed 3 times with PBS, followed by 1% Triton X-100. After standardization to the total protein content, ALP activity was quantified using p-nitrophenyl substrate at 405 nm.

### Alizarin red S (ARS) staining and quantification

The hDPSCs were cultured in an osteogenic induction medium for 14 days and used for ARS staining. ARS staining was performed to visualize the mineral deposition. The cell samples were washed with PBS 3 times and fixed in a solution of 4% paraformaldehyde for 20 min. They were stained with 0.1% ARS (pH = 4.2) for 20 min at room temperature. To detect the quantity of mineralized nodules, the dissolved ARS stain in 10% cetylpyridinium chloride (Sigma-Aldrich) was treated for 60 min, the final absorbance was measured at 570 nm.

### RNA isolation and quantitative real-time polymerase chain reaction (qRT-PCR)

The RNAs were isolated by TRIzol reagent (Ambion). For mRNA detection, a PrimeScript RT reagent kit (Takara) was used for the DNA synthesis, and quantitative real-time PCR was conducted in triplicate with SYBR Premix Ex Taq (Takara). The primers of mRNAs were synthesized by GenePharma and listed in Table S2. For miRNA detection, a miRNA qRT-PCR Detection Kit (GeneCopoeia) was used for the cDNA synthesis and quantitative detection. GAPDH and U6 were used as internal controls for mRNAs and miR-20a-5p, respectively. The relative expression was calculated by the formula 2^-ΔΔ^Ct.

### Western blot

The protein levels of RUNX2, BSP, OPN, BAMBI, Smad5, p38, p-Samd5, and p-p38 were detected by Western blot. Total cell proteins were extracted with radioimmunoprecipitation assay (RIPA) lysis buffer (Sigma). BCA Protein Assay Kit (Beyotime) was used to determine the protein concentration. An equal volume of samples was placed on sodium dodecyl sulfate-polyacrylamide gel electrophoresis (SDS-PAGE) and then transferred onto PVDF membranes (Millipore). Primary antibodies against anti-RUNX2 (1:1000; Abcam), anti-OPN (1:1000; Huabio), anti-BSP (1:1000; Abcam), anti-Smad5 (1:1000; Huabio), anti-p-Smad5 (1:1000; Huabio), anti-p38 (1:1000; Huabio), anti-p-p38 (1:1000; Huabio), and GAPDH (1:1000; Abcam) were incubated with the PVDF membranes at 4°C overnight. The membranes were incubated with secondary antibodies (1:5000; Abcam) for 2 h after washed with TBST. All the protein bands were normalized to GAPDH band.

### Dual-luciferase report assay

Luciferase reporter assays were conducted to demonstrate whether miR-20a-5p targeted BAMBI. BAMBI cDNA fragments with the predicted miR-20a-5p binding sites were amplified and cloned to pEZX-MT06 vector (GeneCopoeia) to construct a wild-type BAMBI (BAMBI-WT) luciferase reporter plasmid. The mutation of miR-20a-5p target sites in the BAMBI 3′ UTR was performed by using a site-directed mutagenesis kit (SBS Genetech) and a mutant-type BAMBI (BAMBI-MU) luciferase reporter plasmid was constructed. The hDPSCs were cotransfected with 100 nM negative control or miR-20a-5p mimics, 1 μg BAMBI-WT or BAMBI-MU luciferase reporter plasmid, and lipofectamine 3000 (Invitrogen). The luciferase activities were determined by the dual-luciferase reporter assay system (Promega), which were standardized to Renilla luciferase and detected by an enzyme-labeled instrument

### Animal surgery

The procedure of animal surgery was approved by the Institutional Review Board of West China Hospital of Stomatology, Sichuan University (WCHSIRB-D-2017-129). We used 32-month-old female nude mice (BALB/c) in this experiment, and each group included ten mice. The calvarial defect (3.5mm) operation was performed in the left parietal bones of mice with a dental drill.

The hDPSCs transfected by NC, miR-20a-5p, and anti-miR-20a-5p were resuspended in 1.2% alginate solution (Sigma) at a density of 5×10^6^ cells per ml in a conical tube, and the suspension was dropped into 1 ml of pre-warmed CaCl_2_ to form the spherical alginate scaffolds which were approximately 3.5mm in diameter. The prepared alginate scaffold with cells was placed in the defect area. After 8 weeks, the skull samples were harvested and fixed in 4% polyoxymethylene for further experiments.

### Micro-computed tomography (micro-CT) analysis

The micro-CT (μCT50; SCANCO) was utilized to determine the new bone formation. Images were captured at the voxel resolution of 10 μm. All the skull samples were in the same scanning tube and scanned with the same parameters. The reconstruction area was set according to the measuring scale and anatomical landmarks. After the files were reconstructed, the bone mineral density (BMD, mg/cm^3^), the ratio of bone volume to tissue volume (BV/TV, %), bone surface (BS, mm^2^), and bone surface density (BS/TV, mm^2^/mm^3^) were calculated using SCANCO Medical Evaluation software.

### Hematoxylin and eosin (H&E) staining and Masson’s trichrome staining

The fixed samples were embedded in paraffin after the thorough decalcification in 10% EDTA for 1 month. The representative sections were cut at 5-μm thickness and stained with H&E as well as Masson’s trichrome. The stained sections were recorded by the light microscope with imaging systems (DS-U3, Nikon, Japan).

### Statistical analysis

The statistical analysis was performed with SPSS software (version 16.0; SPSS, Inc., Chicago, IL, USA). All data were expressed as the means ± standard deviation (SD) of three independent experiments at least. The differences between the two groups were determined by unpaired *t* test, and one-way analysis of variance (ANOVA) was used to analyze differences among more than two groups. *P* value < 0.05 was set as statistical significance.

## Results

### miR-20a-5p was upregulated during the osteogenesis of hDPSCs

**Fig. 1:**
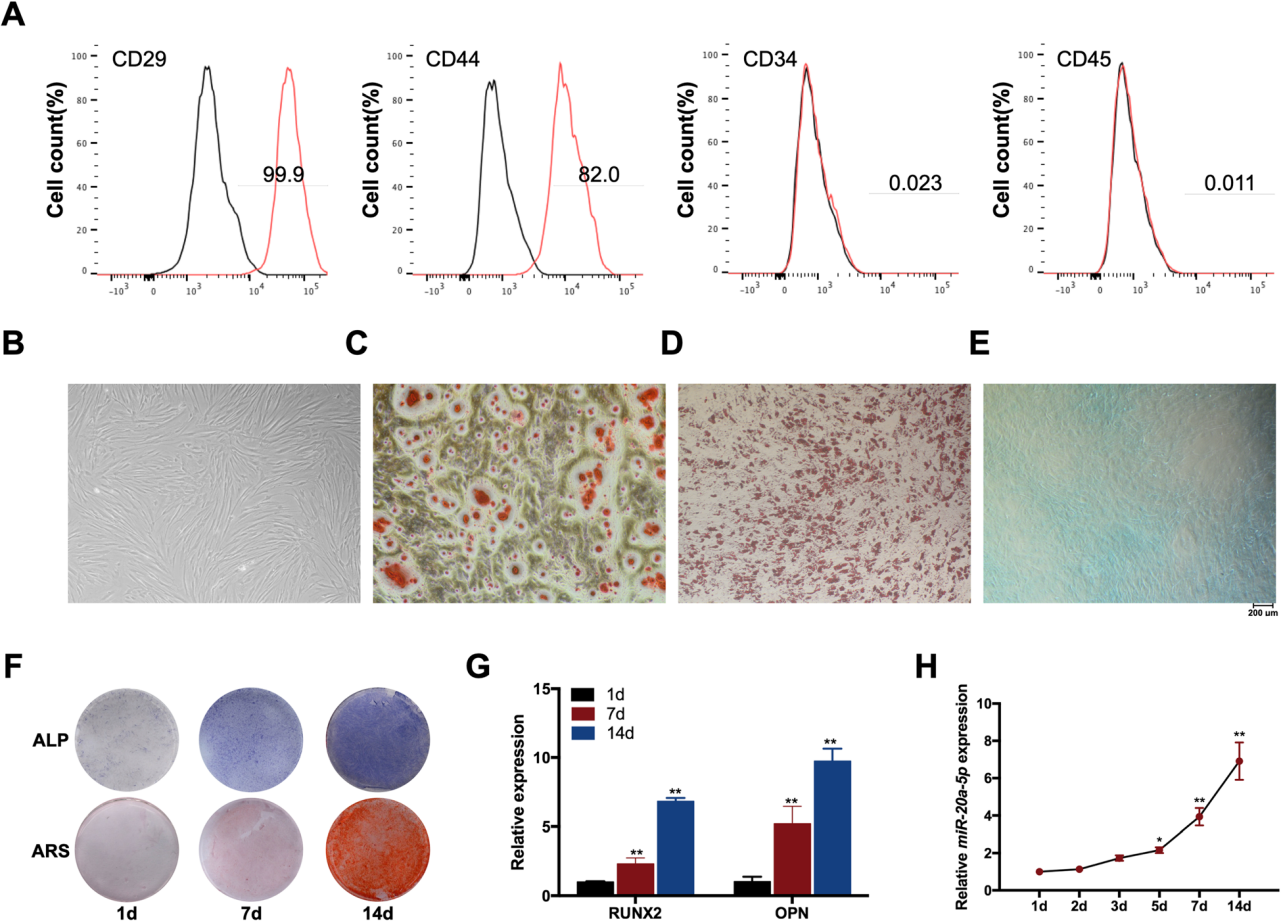
miR-20a-5p was upregulated during the osteogenesis of hDPSCs. **A** hDPSCs were positive for CD29 and CD44, while negative for CD34 and CD45. **B** The morphology of primary hDPSCs was spindle-shaped fibroblast-like. **C**–**E** Osteogenic and chondrogenic differentiations were evaluated by ARS and Alcian Blue staining respectively, and Oil Red O staining was performed for testing adipogenic differentiation. **F** ALP and ARS staining after osteogenic induction for 1, 7, and 14 days. **G** RUNX2 and OPN mRNA levels after osteogenic induction for 1, 7, and 14 days. **H** The expression levels of miR-20a-5p from day 1 to day 14. *p <0.05 and **p <0.01 compared with osteogenic induction for 1 day

The results of flow cytometry revealed that the expression profiles of the MSC markers, CD29 and CD44, were positive, while the hematopoietic markers, CD34 and CD45, were expressed negatively (Fig. 1A). The morphology of primary hDPSCs was spindle-shaped fibroblast-like (Fig. 1B). Osteogenic and chondrogenic differentiations were evaluated by ARS and Alcian Blue staining, respectively, and Oil Red O staining was performed for testing adipogenic differentiation (Fig. 1C–E). The results illustrated that the isolated cells were MSCs.

During osteogenic induction of hDPSCs, ALP and ARS staining results confirmed the typical osteogenesis phenotype, and the results of qRT-PCR showed a significant increased expression of osteogenic markers including RUNX2 and OPN (Fig. 1F–G). The expression of miR-20a-5p was detected during this process to investigate the relationship between miR-20a-5p and osteogenesis of hDPSCs. In general, the expression levels of miR-20a-5p were increased from day 1 to day 14 (Fig. 1H).

### miR-20a-5p facilitated osteogenic differentiation of hDPSCs in vitro

**Fig. 2:**
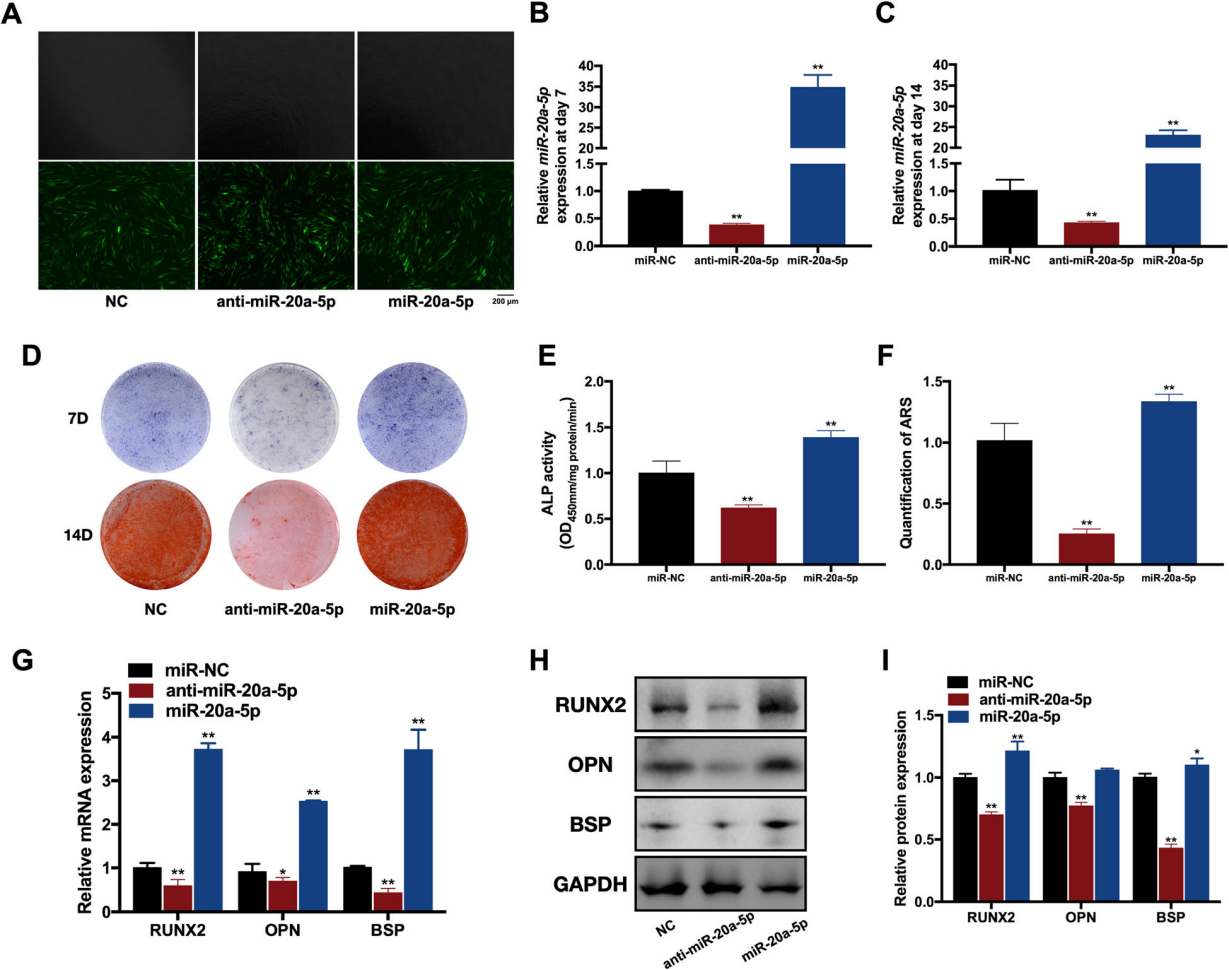
miR-20a-5p facilitated osteogenic differentiation of hDPSCs in vitro. **A** Transfection efficiency in miR-20a-5p, anti-miR-20a-5p, and miR-NC groups. **B** The relative expression of miR-20a-5p in miR-20a-5p, anti-miR-20a-5p, and miR-NC groups at day 7. **C** The relative expression of miR-20a-5p in miR-20a-5p, anti-miR-20a-5p, and miR-NC groups at day 14. **D** ALP staining in miR-20a-5p, anti-miR-20a-5p, and miR-NC groups. **E** ALP activity in miR-20a-5p, anti-miR-20a-5p, and miR-NC groups. **F** Quantification of ARS in miR-20a-5p, anti-miR-20a-5p, and miR-NC groups. **G** The relative mRNA expression levels of RUNX2, OPN, and BSP in miR-20a-5p, anti-miR-20a-5p, and miR-NC groups. **H** Protein levels of RUNX2, OPN, and BSP in miR-20a-5p, anti-miR-20a-5p, and miR-NC groups. **I** Quantification of protein levels of RUNX2, OPN, and BSP in miR-20a-5p, anti-miR-20a-5p, and miR-NC groups. *p <0.05 and **p <0.01 compared with the miR-NC group

To investigate the role of miR-20a-5p in osteogenesis of hDPSCs, we used lentiviral vectors to transfect hDPSCs with miR-20a-5p, anti-miR-20a-5p, and NC lentiviruses. Since GFP was designed as positive markers, the percentage of GFP-positive cells was utilized to calculate the transfection efficiency, which exceeded 80% in three groups (Fig. 2A).

The qRT-PCR was performed to detect the expression of miR-20a-5p on days 7 and 14. The relative expression of miR-20a-5p was over 30-fold higher in the miR-20a-5p group than that in the NC group, while the expression of miR-20a-5p showed a 60% declination in the anti-miR-20a-5p group at day 7 (Fig. 2B). Similarly, the relative expression of miR-20a-5p was 20-fold higher in the miR-20a-5p group than that in the NC group, while the expression of miR-20a-5p showed a 50% declination in the anti-miR-20a-5p group at day 14 (Fig. 2C). These results suggested that the efficiency of gene transfection with lentiviral vectors remained stable in this experiment.

The intensity of ALP staining was stronger in the miR-20a-5p group than that in the NC group, and it was obviously weaker in the anti-miR-20a-5p group (Fig. 2D). Similarly, more calcified nodules were captured in the miR-20a-5p group than those in the NC group (Fig. 2D). Both ALP activity and quantification of ARS were increased in the miR-20a-5p group while were decreased in the anti-miR-20a-5p group (Fig. 2E, F).

The relative mRNA expression levels of RUNX2, OPN, and BSP were upregulated in the miR-20a-5p group, while were downregulated in the anti-miR-20a-5p group significantly (Fig. 2G). Western blot results indicated that protein levels of RUNX2, OPN, and BSP reduced in the anti-miR-20a-5p group, whereas increased in the miR-20a-5p group (Fig. 2H, I).

### miR-20a-5p promoted the regeneration of calvarial defects

To further verify the function of miR-20a-5p on bone formation, miR-20a-5p, anti-miR-20a-5p, and NC lentivirus-treated hDPSCs were loaded on alginate scaffold to fill calvarial defects of nude mice.

**Fig. 3:**
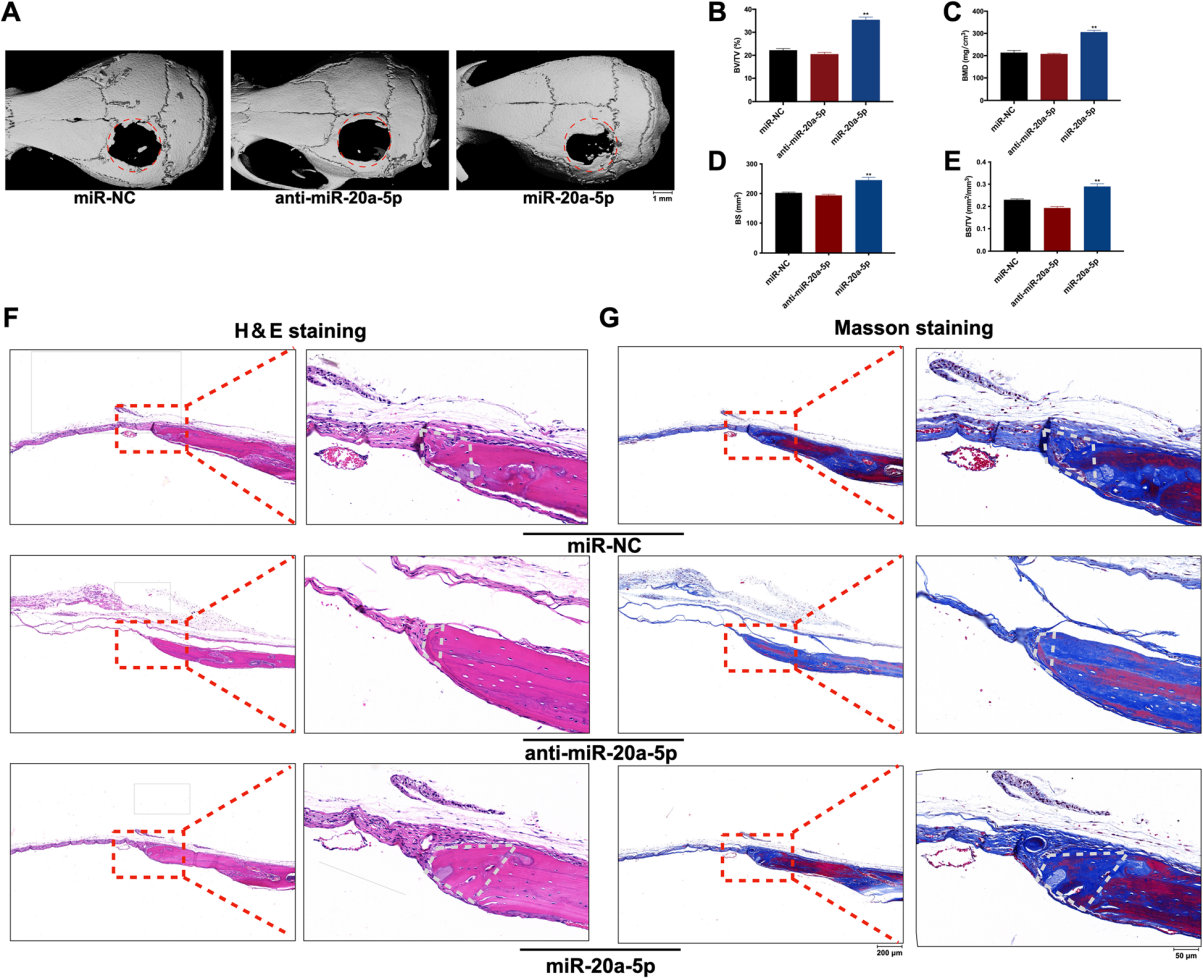
miR-20a-5p promoted the regeneration of calvarial defects. **A** Micro-CT images showed the regeneration of each calvarial defect in miR-20a-5p, anti-miR-20a-5p, and miR-NC groups. **B** BMD in miR-20a-5p, anti-miR-20a-5p, and miR-NC groups. **C** BV/TV in miR-20a-5p, anti-miR-20a-5p, and miR-NC groups. **D** BS in miR-20a-5p, anti-miR-20a-5p, and miR-NC groups. **E** BS/TV in miR-20a-5p, anti-miR-20a-5p, and miR-NC groups. **F** H&E staining illustrated new bone formation in miR-20a-5p, anti-miR-20a-5p, and miR-NC groups. White trapezoid represented new formed bone. **G** Masson’s trichrome staining showed new collagen fibers in miR-20a-5p, anti-miR-20a-5p, and miR-NC groups. White trapezoid represented new formed bone. *p <0.05 and **p <0.01 compared with the miR-NC group

Micro-CT images were used to observe the regeneration of each calvarial defect, illustrating better bone regeneration and smaller defect areas in the miR-20a-5p group compared with the NC group (Fig. 3A). Both BMD and BV/TV showed that new bone formation was much more in the miR-20a-5p group (Fig. 3B, C). Meanwhile, BS and BS/TV demonstrated that miR-20a-5p facilitated more newly generated bone (Fig. 3D, E).

Histological results were consistent with those of micro-CT analyses. The new bone and tissue in H&E staining, which were in blue color in Masson’s trichrome staining, were observed surrounding the bone defects in all groups. Nearly no observable bone formation was found in the anti-miR-20a-5p group, and the sizes of the new bone were relatively larger in the miR-20a-5p group with sufficient aligned collagen fibers than other two groups (Fig. 3F, G).

### BAMBI was the target of miR-20a-5p

**Fig. 4:**
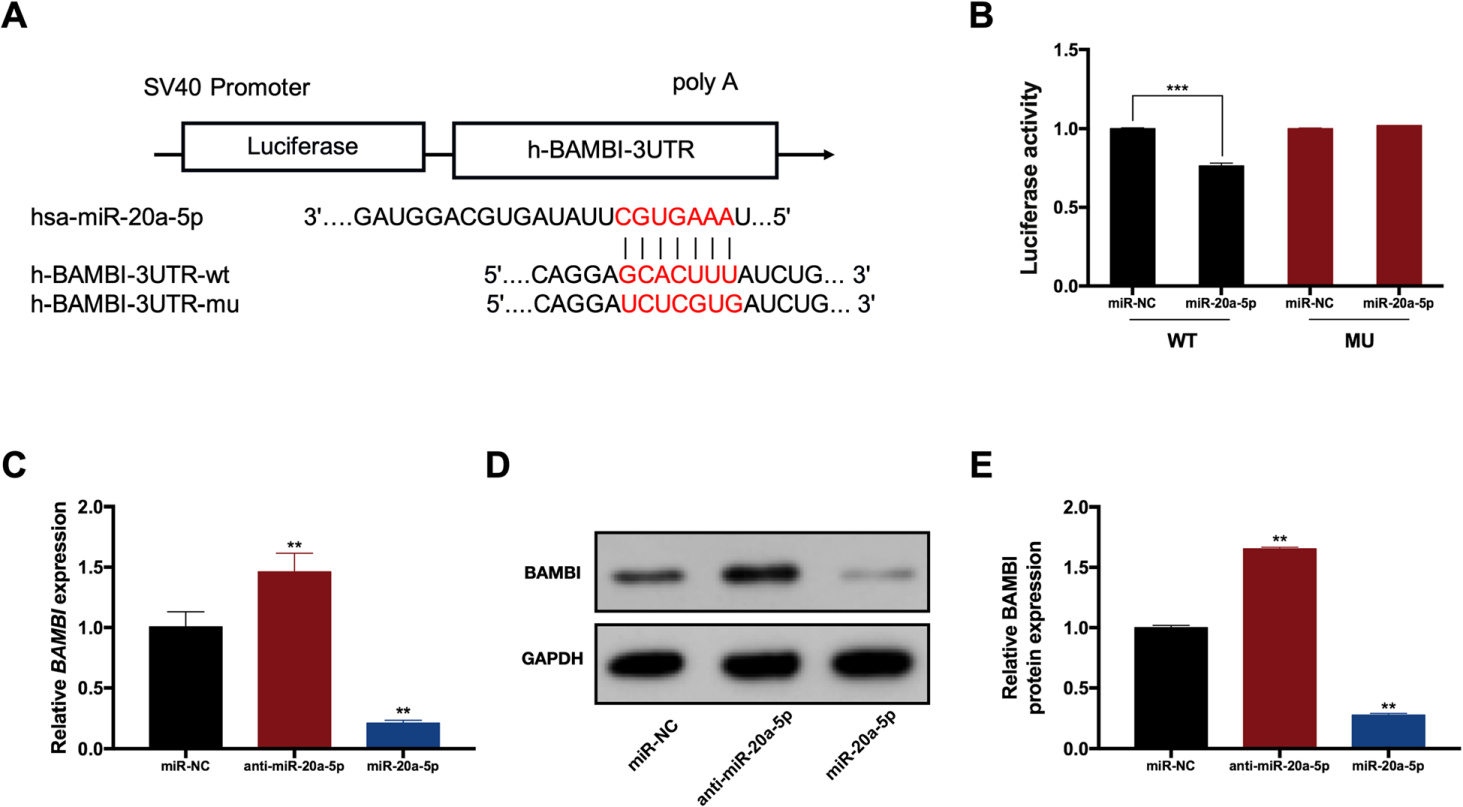
BAMBI was the target of miR-20a-5p. **A** The binding sites between miR-20a-5p and BAMBI in dual-luciferase reporter assay. **B** Luciferase activity in WT and MU groups. **C** The mRNA expression of BAMBI in miR-20a-5p, anti-miR-20a-5p, and miR-NC groups. **D** The protein levels of BAMBI in miR-20a-5p, anti-miR-20a-5p, and miR-NC groups. **E** Quantification of protein levels of BAMBI in miR-20a-5p, anti-miR-20a-5p, and NC groups. *p <0.05 and **p <0.01 compared with the miR-NC group

A dual-luciferase reporter assay was conducted to explore whether miR-20a-5p could bind to BAMBI directly. The wild-type (WT) reporter included a wild 3′-UTR of BAMBI. The mutant-type (MU) reporter included a mutant 3′-UTR with mutated sequences of the miR-20a-5p binding site (Fig. 4A). Overexpression of miR-20a-5p attenuated the luciferase activity in the WT group significantly, while it failed to impact on the luciferase activity in the MU group (Fig. 4B). The mRNA and protein expression levels of BAMBI were increased in the anti-miR-20a-5p group, whereas were decreased in the miR-20a-5p group (Fig. 4C–E).

### miR-20a-5p regulated osteogenesis via targeting BAMBI

**Fig. 5:**
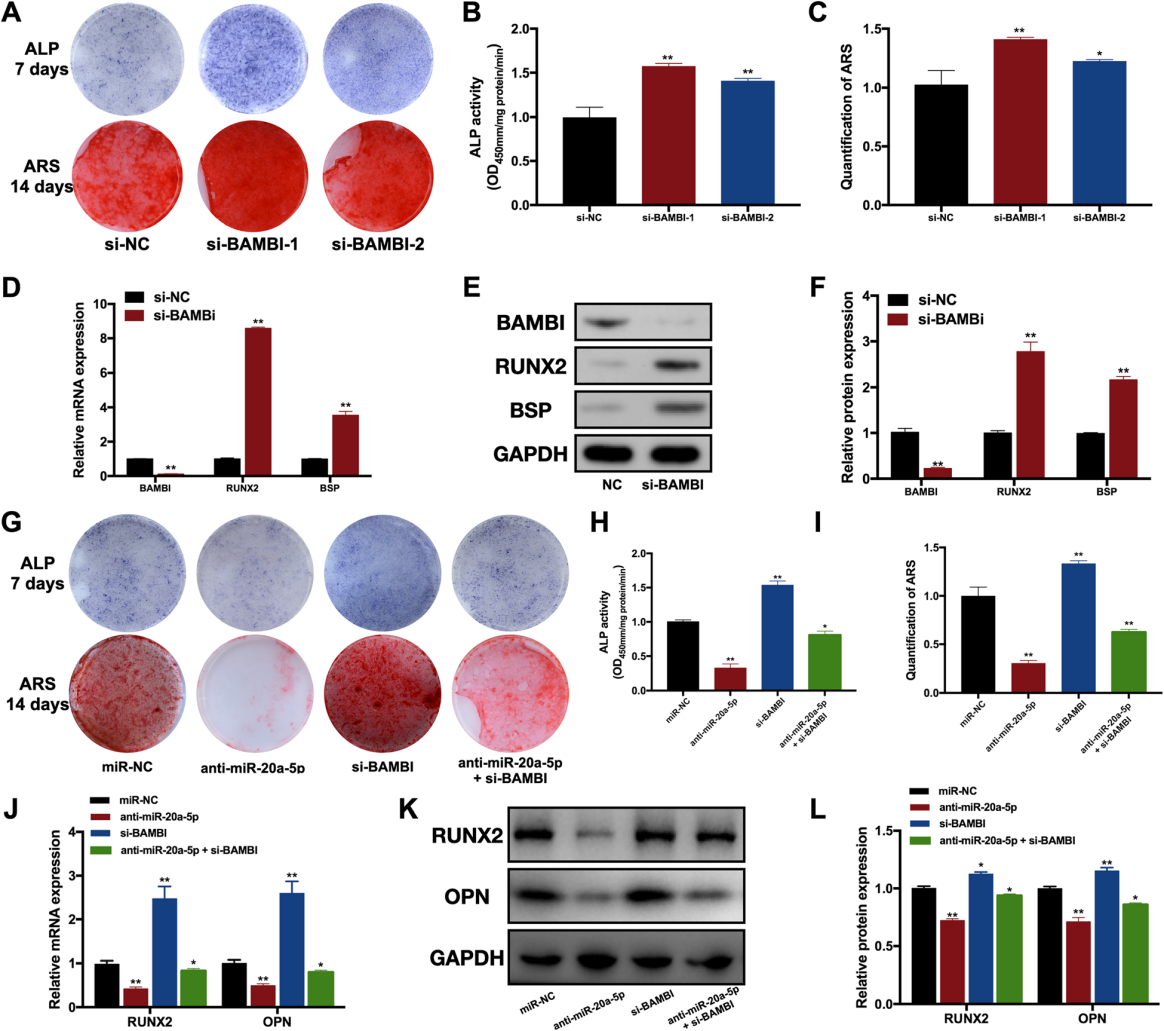
miR-20a-5p regulated osteogenesis via targeting BAMBI. **A** ALP and ARS staining in si-NC and si-BAMBI groups. **B** ALP activity in si-NC and si-BAMBI groups. **C** Quantification of ARS in si-NC and si-BAMBI groups. **D** The relative mRNA expression levels of RUNX2 and BSP in si-NC and si-BAMBI groups. **E** Protein levels of RUNX2 and BSP in si-NC and si-BAMBI groups. **F** Quantification of protein levels of RUNX2 and BSP in si-NC and si-BAMBI groups. **G** ALP and ARS staining in miR-NC, anti-miR-20a-5p, si-BAMBI, and anti-miR-20a-5p plus si-BAMBI groups. **H** ALP activity in miR-NC, anti-miR-20a-5p, si-BAMBI, and anti-miR-20a-5p plus si-BAMBI groups. **I** Quantification of ARS in miR-NC, anti-miR-20a-5p, si-BAMBI, and anti-miR-20a-5p plus si-BAMBI groups. **J** The mRNA expressions of RUNX2 and OPN in miR-NC, anti-miR-20a-5p, si-BAMBI, and anti-miR-20a-5p plus si-BAMBI groups. **K** The protein levels of RUNX2 and OPN in miR-NC, anti-miR-20a-5p, si-BAMBI, and anti-miR-20a-5p plus si-BAMBI groups. **L** Quantification of protein levels of RUNX2 and OPN in miR-NC, anti-miR-20a-5p, si-BAMBI, and anti-miR-20a-5p plus si-BAMBI groups. *p <0.05 and **p <0.01 compared with the si-NC/miR-NC groups

The intensity of ALP staining was stronger, and more calcified nodules were captured in si-BAMBI groups (Fig. 5A). ALP activity and quantification of ARS were also increased in si-BAMBI groups (Fig. 5B, C). The relative mRNA and protein expression levels of RUNX2 and BSP were upregulated in si-BAMBI groups (Fig. 5D–F).

To investigate whether miR-20a-5p regulated osteogenesis of hDPSCs via BAMBI, we performed cell co-transfection including anti-miR-20a-5p and si-BAMBI. After osteogenic induction for 7 and 14 days, si-BAMBI partially reversed the suppression effect of anti-miR-20a-5p on osteogenesis according to ALP and ARS staining (Fig. 5G–I). As for the mRNA and protein levels of osteogenic markers, si-BAMBI restored the downregulation of RUNX2 and OPN (Fig. 5J–L).

### miR-20a-5p/BAMBI axis-regulated osteogenesis via phosphorylation of Smad5 and p38

**Fig. 6:**
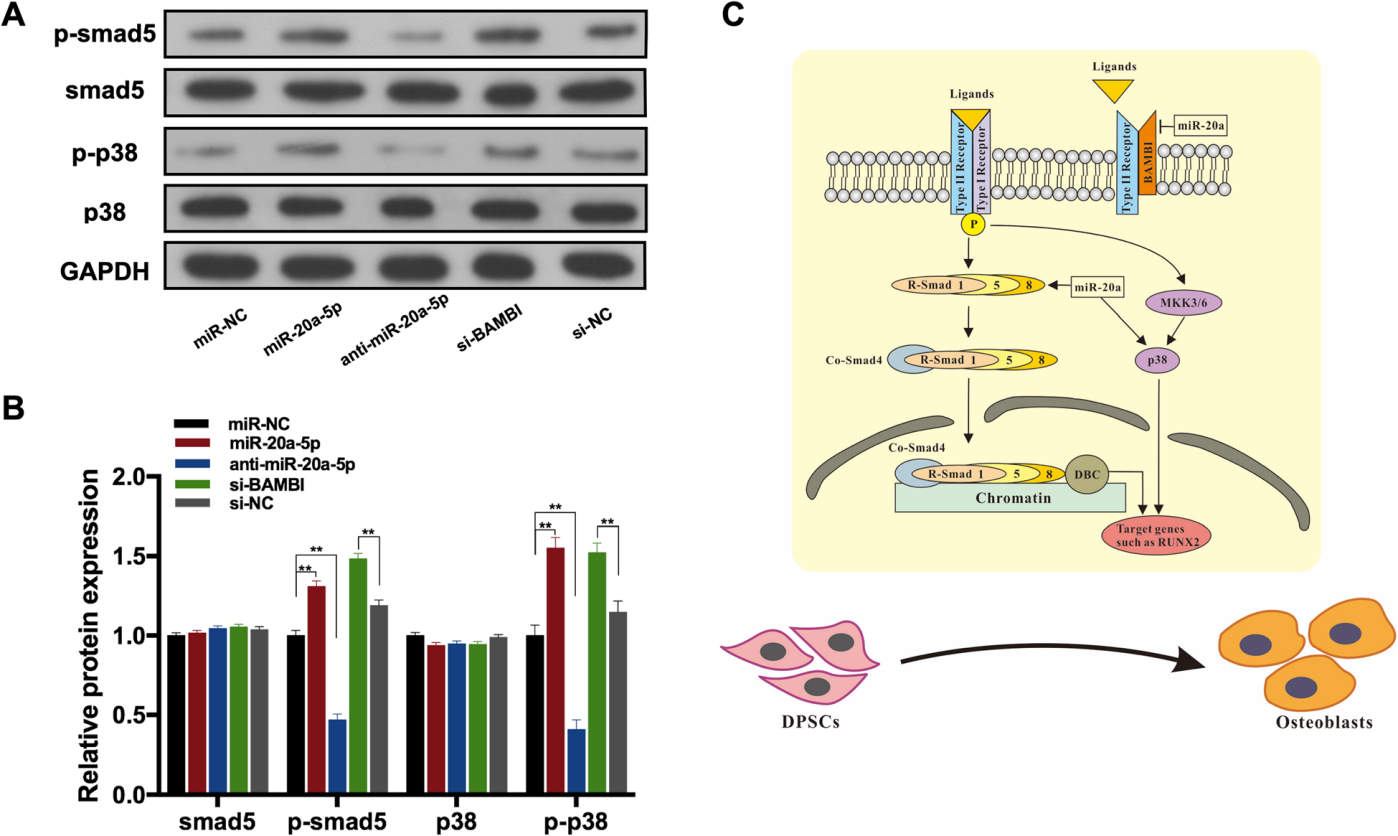
miR-20a-5p/BAMBI axis-regulated osteogenesis via phosphorylation of Smad5 and p38. **A** Western blot analyses of pSmad5, Smad5, p-p38, p38, and the internal control GAPDH in miR-NC, miR-20a-5p, anti-miR-20a-5p, si-BAMBI, and si-NC groups. **B** The protein levels of pSmad5, Smad5, p-p38, p38, and GAPDH in miR-NC, miR-20a-5p, anti-miR-20a-5p, si-BAMBI, and si-NC groups. **C** Schematic model showed that miR-20-5p promotes osteogenic differentiation by the BAMBI-mediated activation of p-Smad5 and p-p38 pathways. **p <0.01 compared with the si-NC/miR-NC groups

In this study, p-Smad5 was downregulated in anti-miR-20a-5p significantly, while increased in the miR-20a-5p group and si-BAMBI groups (Fig. 6A, B). Phosphorylation of p38 (p-p38) was decreased in anti-miR-20a-5p significantly, whereas increased in the miR-20a-5p group and si-BAMBI groups (Fig. 6A, B). These results demonstrated that miR-20a-5p/BAMBI axis may regulate osteogenesis of hDPSCs by activating phosphorylation of Smad5 and p38. The schematic model illustrated that miR-20-5p promotes osteogenic differentiation by the BAMBI-mediated activation of p-Smad5 and p-p38 pathways (Fig. 6C).

## Discussion

The hDPSCs have the advantages of lower immunogenicity and superior multilineage differentiation potential and self-renewal ability. The hDPSCs are isolated from dental pulp, which is a noninvasive and safer method without ethical controversy. They have been considered ideal candidates in stem cell and bone regenerative therapies [28–32]. Therefore, it is important to clarify the mechanisms of osteogenic differentiation of hDPSCs.

As a member of the miR-17~92 gene cluster, miR-20-5p has been found to participate in a variety of cellular activities. The majority of current studies focused on the tumor proliferation and development. LncRNA PVT1 could promote the development of pancreatic ductal adenocarcinoma by serving as a sponge to miR-20-5p [21]. Similarly, long non-coding RNA SNHG16 was found to promote the glioma malignancy by binding miR-20-5p competitively [33]. Additionally, miR-20-5p could target ribonucleotide reductase subunit M2 (RRM2) to regulate the gemcitabine chemosensitivity in pancreatic cancer cells and enhance the growth of triple-negative breast cancer (TNBC) cells by targeting Runx3, Bim, and p21 [20, 22]. These findings demonstrated the essential roles of miR-20a-5p in regulating carcinogenesis in different types of tumors. However, the function of miR-20a-5p on osteogenesis was rarely studied, and only two studies reported that miR-20a-5p could regulate the osteogenic differentiation in hMSCs and hASCs, respectively [24, 34].

In this study, we isolated and cultured hDPSCs successfully and then confirmed their potential for osteogenic differentiation. During the osteogenic induction of hDPSCs, the expression level of miR-20a-5p is upregulated, which indicated that miR-20a-5p may serve positive roles in this process. After cell transduction, hDPSCs in the miR-20a-5p group showed increased ALP activity, stronger extracellular matrix mineralization, and upregulated gene expression of RUNX2 and OPN. Additionally, the regeneration of calvarial defects was enhanced in the miR-20a-5p group. Conversely, relevant results in anti-miR-20a-5p showed a reverse trend. These findings confirmed the positive roles of miR-20a-5p in the osteogenesis of hDPSCs.

BAMBI is a transforming growth factor β (TGF-β) pseudoreceptor, which is structurally similar to TGF-β-family type I receptors but without the intracellular kinase domain. BAMBI could interact with TGF-β receptors to inhibit the formation of receptor complexes and then prevent downstream signaling [35]. Several studies reported the relationship between BAMBI and members of miR-17~92 gene cluster [24, 36–38]. In our study, the dual-luciferase reporter assay validated that BAMBI was a target of miR-20a-5p, which correlated well with the results of qRT-PCR and Western blot. We found that silencing BAMBI partially reversed the suppression effect of anti-miR-20a-5p on osteogenesis of DPSCs, suggesting BAMBI could block the suppression effect of anti-miR-20a-5p on osteogenesis. Therefore, we hypothesized that miR-20a-5p might downregulate BAMBI to activate ligand-receptor complexes, which could promote the osteogenesis of hDPSCs via TGF-β signaling pathway.

In canonical TGF-β signaling, the activation of receptor complexes could phosphorylate intracellular receptor-activated Smad (R-Smad) proteins, including Smad1, Smad5, and Smad8. Subsequently, the phosphorylated Smad1/5/8 could bind with the common-mediator Smad (Co-Smad), Smad4, to form a complex translocating into the nuclei and activating the expression of corresponding targets to modulate osteogenic genes expression [39, 40]. Our study showed that p-Smad5 was downregulated significantly when miR-20a-5p was silenced, while the expression level of p-Smad5 was increased when miR-20a-5p was overexpressed or BAMBI was silenced. It suggested that miR-20a-5p may activate p-Smad5 to regulate osteogenesis of hDPSCs, which might be mediated by BAMBI. Additionally, TGF-β receptor complex activation could activate p38-mitogen-activated protein kinase (p38-MAPK) signaling cascade to regulate transcriptional responses. It is showed that the p38-MAPK pathway actives the phosphorylation of Osterix and Runx2 to regulate MSC differentiation positively [41]. p38 also performs negative feedback on canonical TGF-β-Smad signaling, which leads to tight regulated networks of osteogenesis [42]. Our study showed that phosphorylation of p38 was decreased when miR-20a-5p was silenced, whereas p-p38 was upregulated when miR-20a-5p was overexpressed or BAMBI was silenced. These results demonstrated that miR-20a-5p/BAMBI axis could also regulate osteogenesis of hDPSCs via p-p38.

## Conclusion

In conclusion, this study was the first to reveal the function of miR-20a-5p during osteogenesis of hDPSCs. MiR-20a-5p was showed to participate in osteogenesis of hDPSCs both in vitro and vivo. It is suggested that miR-20a-5p regulated BAMBI to activate the phosphorylation of Smad5 and p38, indicating miR-20a-5p orchestrated osteogenic differentiation of hDPSCs through Smad and non-Smad signaling. These findings provide a novel miRNA-related therapeutic strategy for bone regeneration via hDPSCs.

## Supplementary information

**Additional file 1:.** Table S1. Sequences of RNA oligoribonucleotide. Table S2. T Primers for quantitative expression analysis of qRT-PCR.

## Data Availability

The datasets used during the current study are available from the corresponding authors on reasonable request.

## Abbreviations

ALP: Alkaline
ARS: Alizarin red S
BAMBI: Bone morphogenetic protein and activin membrane-bound inhibitor
BMD: Bone mineral density
BV/TV: The ratio of bone volume to tissue volume
Co-Smad: Common-mediator Smad
DMEM: Dulbecco’s modified Eagle’s medium
GFP: Green fluorescent protein
hASCs: Human adipose-derived stem cells
H&E: Hematoxylin and eosin
hDPSCs: Human dental pulp stem cells
micro-CT: Micro-computed tomography
miRNAs: MicroRNAs
MSCs: Mesenchymal stem cells
NC: Negative control
PBS: Phosphate-buffered saline
p38-MAPK: p38-mitogen-activated protein kinase
qRT-PCR: Quantitative reverse transcription-polymerase chain reaction
RIPA: Radio immunoprecipitation assay
RRM2: Ribonucleotide reductase subunit M2
R-Smad: Receptor-activated Smad
SDS-PAGE: Sodium dodecyl sulfate-polyacrylamide gel electrophoresis
siRNAs: Small interfering RNAs
TNBC: Triple-negative breast cancer
TGF-β: Transforming growth factor β
