# Correction: miR-20a-5p contributes to osteogenic differentiation of human dental pulp stem cells by regulating BAMBI and activating the phosphorylation of Smad5 and p38

**DOI:** 10.1186/s13287-022-02870-8

**Published:** 2022-04-29

**Authors:** Xiao Cen, Xuefeng Pan, Bo Zhang, Wei Huang, Fang Pei, Tao Luo, Xinqi Huang, Jun Liu, Zhihe Zhao

**Affiliations:** 1 State Key Laboratory of Oral Diseases & National Clinical Research Center for Oral Diseases, West China Hospital of Stomatology, Sichuan University, No. 14, 3rd Section, South Renmin Road, Chengdu, 610041 Sichuan People’s Republic of China; 2 Department of Temporomandibular Joint, West China Hospital of Stomatology, Sichuan University, Chengdu, People’s Republic of China; 3 Department of Orthodontics, West China Hospital of Stomatology, Sichuan University, Chengdu, People’s Republic of China; 4 Department of Stomatology, Sichuan Cancer Hospital & Institute, Sichuan Cancer Center, School of Medicine, University of Electronic Science and Technology of China, Chengdu, People’s Republic of China

## Correction to: Stem Cell Research & Therapy (2021) 12:421 https://doi.org/10.1186/s13287-021-02501-8

Following publication of the original article [1], the authors have identified that the incorrect image of ALP staining for 14d in Fig. 1F were included due to an error during manuscript typesetting. The corrected image of ALP staining for 14d has been updated in Fig. 1F.

**Fig. 1:**
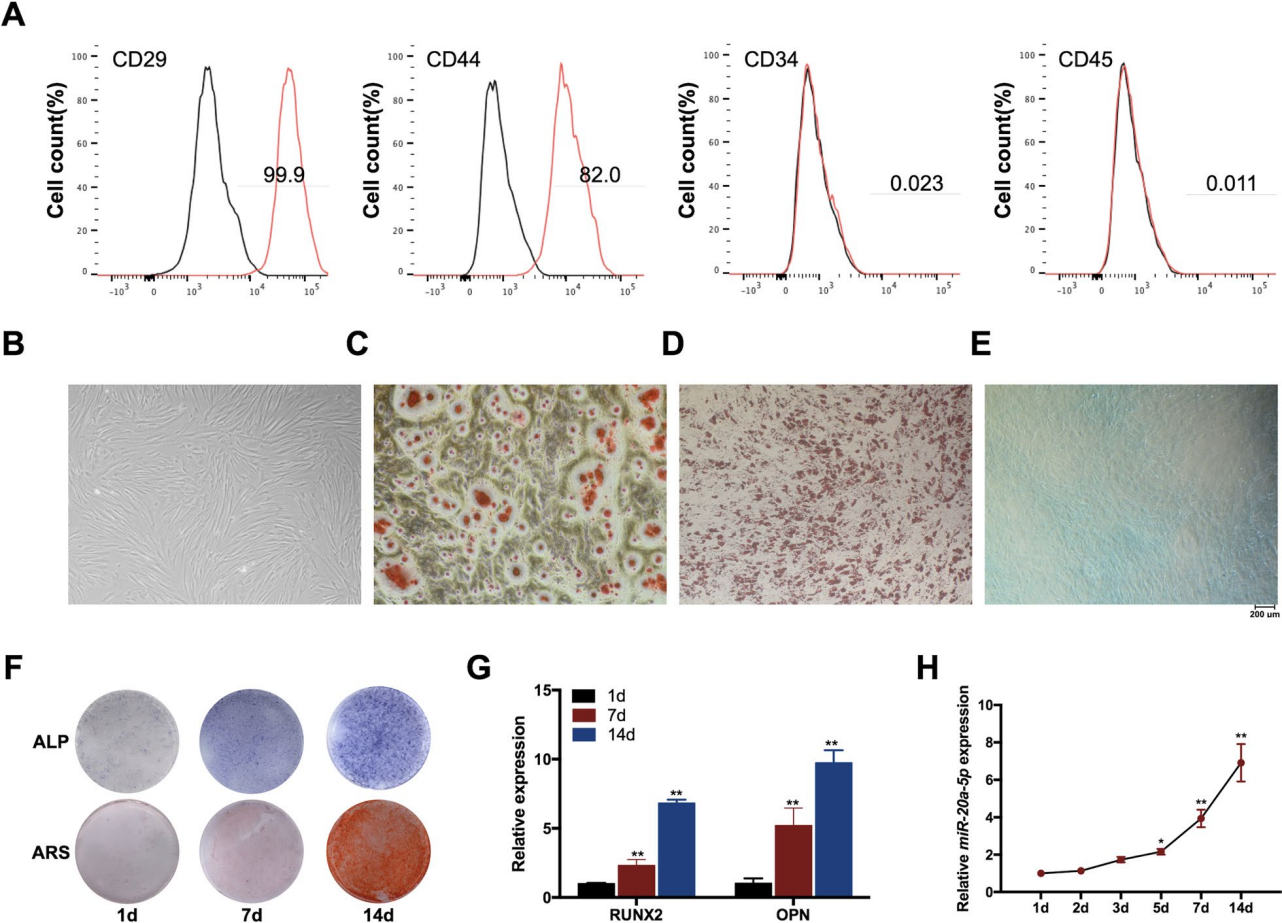
miR-20a-5p was up-regulated during the osteogenesis of hDPSCs. **A** hDPSCs were positive for CD29 and CD44, while negative for CD34 and CD45. **B** The morphology of primary hDPSCs was spindle-shaped fibroblast-like. **C**–**E** Osteogenic and chondrogenic differentiations were evaluated by ARS and Alcian Blue staining respectively, and Oil Red O staining was performed for testing adipogenic differentiation. **F** ALP and ARS staining after osteogenic induction for 1, 7, and 14 days. **G** RUNX2 and OPN mRNA levels after osteogenic induction for 1, 7, and 14 days. **H** The expression levels of miR-20a-5p from day 1 to day 14. *p < 0.05 and **p < 0.01 compared with osteogenic induction for 1 day

Therefore, the revised Fig. 1 is given in this article.
