# Supplementary material for: miR-20a-5p contributes to osteogenic differentiation of human dental pulp stem cells by regulating BAMBI and activating the phosphorylation of Smad5 and p38
Source: Stem Cell Res Ther. 2021 Jul 22;12:421. doi: 10.1186/s13287-021-02501-8 (PMC8296686; doi:10.1186/s13287-021-02501-8)
Supplement: Supplementary file 1 — Additional file 1:. Table S1. Sequences of RNA oligoribonucleotide. Table S2. T Primers for quantitative expression analysis of qRT-PCR. [file 13287_2021_2501_MOESM1_ESM.docx]

**TABLE S1** The sequences of the RNA oligoribonucleotide

| Name | Sequences (5’-3’) |
| --- | --- |
| miR-20a-5p mimic | CAGCTTCTGTAGCACTAAAGTGCTTATAGTGCAGGTAGTGTGT  CGTCATCTACTGCATTACGAGCACTTACAGTACTGCCAGCTG |
| miR-20a-5p inhibitor | CTACCTGCACTATAAGCACTTTA |
| miR-NC | TTCTCCGAACGTGTCACGT |
| si-BAMBi | F: UGCCACUCCAGCUACAUCUUTT  R: AAGAUGUAGCUGGAGUGGCTT |
| si-NC | F: UUCUCCGAACGUGUCACGUTT  R: ACGUGACACGUUCGGAGAATT |

**TABLE S2** Primer sequences list of qRT-PCR

| Name | Primer sequences (5’-3’) |
| --- | --- |
| miR-20a-5p | F: CGGCGCTAAAGTGCTTATAGTGC  R: ATCCAGTGCAGGGTCCGAGG |
| U6 | F: GCTTCGGCAGCACATATACTAAAAT  R: CGCTTCACGAATTTGCGTGTCAT |
| RUNX2 | F: CTACTATGGCACTTCGTCAGGAT  R: ATCAGCGTCAACACCATCATT |
| OPN | F: GGCTAAACCCTGACCCATCTC  R: GTCAATGGAGTCCTGGCTGTC |
| BSP | F: GGCGACACTTACCGAGCTTA  R: GGGGGCTTCACTGATGGTAG |
| BAMBI | F: GGTCATTGCCGTGCCCATT  R: CATCTGTTGCCGCTGATCCTG |
| GAPDH | F: GGCCTCCAAGGAGTAAGACC  R: AGGGGAGATTCAGTGTGGTG |
